# Interaction Between Autophagy and *Porphyromonas gingivalis*-Induced Inflammation

**DOI:** 10.3389/fcimb.2022.892610

**Published:** 2022-07-01

**Authors:** Sen Kang, Anna Dai, Huiming Wang, Pei-Hui Ding

**Affiliations:** Stomatology Hospital, School of Stomatology, Zhejiang University School of Medicine, Clinical Research Center for Oral Diseases of Zhejiang Province, Key Laboratory of Oral Biomedical Research of Zhejiang Province, Zhejiang, China

**Keywords:** autophagy, *Porphyromonas gingivalis*, inflammation, xenophagy, macroautophagy

## Abstract

Autophagy is an immune homeostasis process induced by multiple intracellular and extracellular signals. Inflammation is a protective response to harmful stimuli such as pathogen microbial infection and body tissue damage. *Porphyromonas gingivalis* infection elicits both autophagy and inflammation, and dysregulation of autophagy and inflammation promotes pathology. This review focuses on the interaction between autophagy and inflammation caused by *Porphyromonas gingivalis* infection, aiming to elaborate on the possible mechanism involved in the interaction.

## Introduction

Periodontal disease (PD) is one of the most common chronic inflammatory oral diseases. It ranks as the sixth most prevalent disease worldwide (Kassebaum et al., 2017). PD can be mainly divided into two major categories: gingivitis and periodontitis. Periodontitis is the primary cause of tooth loss in adults. PD is initiated by gram-negative microorganisms in the sub-gingival plaque biofilms (Socransky et al., 1998). Among these microorganisms, *Porphyromonas gingivalis* (*P. gingivalis*) is a keystone pathogenic bacterium (Whitney et al., 1992; How et al., 2016; Ebersole et al., 2020). However, recent reports have suggested that periodontitis might raise the risk of several systematic inflammatory diseases, such as cardiovascular diseases (Humphrey et al., 2008), inflammatory bowel diseases (Jia et al., 2020), rheumatoid arthritis (Rutger Persson, 2012), Alzheimer’s disease (Singhrao et al., 2015), hepatitis (Yoneda et al., 2012) and *et al*. The links between them probably depends on the *P. gingivalis*’ ability of adhering and internalizing to the host cells (Hajishengallis and Lamont, 2014).

Autophagy is a self-degradative process. It is important in balancing energy and maintaining homeostasis in response to various stimuli, including bacterial infection (Krause et al., 2018), hypoxia (Cosin-Roger et al., 2017), reactive oxygen species (ROS) (Li et al., 2016), and endoplasmic reticulum (ER) stress (Periyasamy et al., 2016). Cellular contents such as microorganisms or damaged organelles can be delivered into lysosomes for degradation so that nutrients can be recycled by autophagy (Mizushima et al., 2008; Mizushima and Komatsu, 2011).

Autophagy has been linked with immune responses to defense against infection (Randow and Munz, 2012; Thurston et al., 2016). The type of antibacterial autophagy (xenophagy) can specifically target intracellular pathogens to lysosomes, and the involved mechanism relies on the cargo receptors, galectin 8 and ubiquitin (Thurston et al., 2012; Ravenhill et al., 2019). Autophagy plays a protective role in decreasing *P. gingivalis*-induced inflammation (Bullon et al., 2012; Park et al., 2017). When host cells are stimulated by *P. gingivalis* or its virulence factors, autophagy can inhibit the activation of inflammasomes such as NLR family pyrin domain containing 3 (NLRP3) and the secretion of pro-inflammatory cytokines such as IL-1β through degrading endogenous stimulus, negatively regulating inflammation and establishing cellular homeostasis (Harris et al., 2011; Takahama et al., 2018).

However, bacteria such as *P. gingivalis* have evolved a variety of strategies to survive in the host cells. For example, *P. gingivalis* can enter single membrane vesicles and prevent autophagosome fusion with the lysosome. In this way, *P. gingivalis can* utilize proteins or other nutrients in the autophagosome to provide energy for its survival (Belanger et al., 2006; Lee et al., 2018). The interaction between autophagy and bacterial infection and how it affects the immune response in host cells is unclear. This review focuses on the studies within the last five years on the interaction between autophagy, *P. gingivalis* infection, and inflammation and summarizes the possible involved mechanisms.

## Autophagy Introduction

Autophagy can be divided into macroautophagy, chaperone-mediated autophagy (CMA), and microautophagy. Only macroautophagy involves the formation of another organelle termed autophagosome (Yim and Mizushima, 2020). This review focuses on macroautophagy. Macroautophagy is divided into selective and non-selective autophagy, depending on whether the substrate is specifically targeted to the autophagosome (Kissova et al., 2007).

### Non-Selective Autophagy Mechanisms

**Figure 1:**
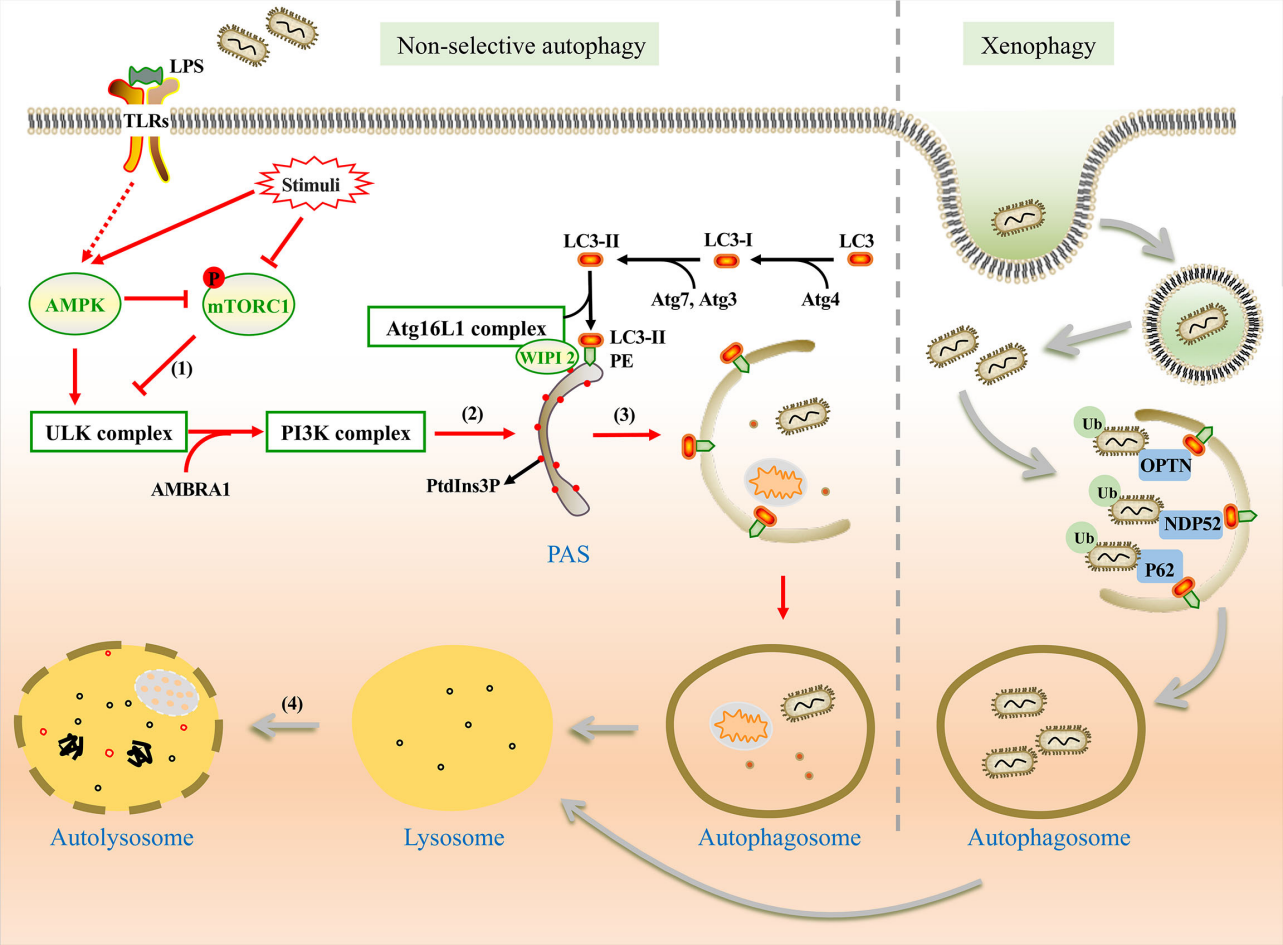
The mechanism of non-selective and xenophagy. Non-selective autophagy (Left) is initiated by the stimulation of LPS. (1) Under the stimulation, AMPK is activated and mTORC1 is inhibited. Thus, the ULK1 complex is activated and phosphorylates the PI3K complex. (2) WIPI2 recruits the Atg16L1 complex to the pre-autophagosomal structure (PAS) by the PtdIns3P binding protein and promotes the conjugation between LC3-II and phosphatidylethanolamine (PE). (3) Autophagic membrane is expanded and completed. Damaged organelles, pathogens, and other components are encapsulated in autophagosomes without selection. (4) Autophagosome fuses with lysosome for degradation of the substances in vesicles. Xenophagy (Right) can capture bacteria selectively. With the help of ubiquitin-binding protein adaptors, such as p62, OPTN, and NDP52, ubiquitinated bacteria are captured by autophagosome for degradation.

Non-selective autophagy is a non-specific process during which cytoplasmic components are recycled into autolysosomes for degradation and damaged organelles are removed under stress conditions to maintain cellular homeostasis. This type of autophagy involves a series of steps: initiation, nucleation, formation of the phagophore, phagophore elongation, fusion of the autophagosome with lysosomes, and degradation (Feng et al., 2014) (**Figure 1**). The formation of these structures is regulated by many signal pathways and related proteins are encoded by autophagy-related genes (ATGs). The process involves two complexes, the unc-51-like kinase (ULK) complex and class III phosphatidylinositol 3-kinase (PI3K) complex. It also includes two ubiquitin-like conjugations (UBL) systems, the Atg16L1 complex and microtubule-associated protein 1 light chain 3 (LC3) (Mizushima and Komatsu, 2011; Mizushima and Levine, 2020).

The upstream regulators of non-selective autophagy are the mammalian target of rapamycin complex 1 (mTORC1) and adenosine monophosphate-activated protein kinase (AMPK). Under nutrient-rich conditions, mTORC1 inhibits autophagy by phosphorylating ULK1 (Ganley et al., 2009). However, as a positive regulator, AMPK can directly activate autophagy by phosphorylating ULK1 at specific sites or indirectly activate autophagy by impairing mTORC1 (Yang and Klionsky, 2010; Fan et al., 2015).

Under stress conditions, autophagy is initiated by the assembly of the ULK complex which phosphorylates Beclin-1-regulated autophagy protein 1 (AMBRA1) and leads to the activation of the PI3K complex (Li et al., 2016). PI3K complex promotes the formation of phosphatidylinositol-3-phosphate (PtdIns3P) on the endoplasmic reticulum (ER), Golgi intermediate, or other membrane structures to mediate membrane nucleation (Maho et al., 2013). The Atg16L1 complex is recruited to the pre-autophagosomal structure (PAS) by PtdIns3P binding protein such as WIPI2 (Polson et al., 2010; Dooley et al., 2014). LC3 is processed into LC3-II with the assistance of Atg7, Atg4, and Atg3 (Glick et al., 2010), and then LC3-II binds with phosphatidylethanolamine (PE), which is essential for the expansion and completion of the autophagic membrane (Mizushima et al., 1998; Glick et al., 2010). Finally, the autophagosome fuses with the lysosome to form an autolysosome with the involvement of some proteins, including the small GTPase Rab7, syntaxin 17, vesicle-associated membrane protein (VAMP7, VAMP8) (Jager et al., 2004; Itakura et al., 2012; Wang et al., 2016).

### Selective Autophagy (Xenophagy Mechanisms)

Evidence indicates that selective autophagy can degrade specific proteins, organelles, and invading bacteria. According to the specific targeted substrates, selective autophagy can be classified as xenophagy (Huang and Brumell, 2014; Castrejon-Jimenez et al., 2015), mitophagy (Zhong et al., 2016), ER-phagy (Bernales et al., 2006), and lipophagy (Singh et al., 2009). Among them, xenophagy acts as anti-bacterial autophagy that plays a crucial role in eliminating intracellular bacterial (Wang and Li, 2020).

Unlike nutrient starvation-induced non-selective autophagy, ubiquitination is more important in xenophagy rather than the regulation of mTORC1 and AMPK (Shaid et al., 2013; Losier et al., 2019). Ubiquitinated bacteria could be captured by ubiquitin-binding protein adaptors such as sequestosome 1 (SQSTM1/p62) (Zheng et al., 2009), nuclear domain 10 protein 52 (NDP52) (Li et al., 2013), optineurin (OPTN) (Wild et al., 2011), and et al. These adaptors interact with LC3, which promotes the formation of autophagosome (Sharma et al., 2018; Wu and Li, 2019). Similar to non-selective autophagy, the fate of autophagosomes is to fuse with lysosomes for degradation (**Figure 1**).

It is supposed that *P. gingivalis* and its virulence factors are tagged with ubiquitin, which allows them to bind to the adaptor proteins NDP52 and p62 (Lee et al., 2018). These adaptor proteins bind to LC3, thereby ensuring the bacteria be tethered and trapped in the autolysosome.

## *P. gingivalis* Introduction

*P. gingivalis* expresses diverse virulence factors, such as lipopolysaccharide (LPS), fimbriae, capsule, gingipains, haemagglutinins, and other surface structures (How et al., 2016). Among them, *P. gingivalis* LPS and fimbriae are two special virulence factors in triggering inflammation, for their structure is variable. LPS consists of three parts: lipid A, core oligosaccharide, and O-antigen. Among them, lipid A is the core component and effector of LPS (Ogawa and Yagi, 2010). *P. gingivalis* expresses two forms of fimbriae, including long fimbriae, and short fimbriae. Long fimbriae contain FimA subunits protein and short fimbriae are comprised of Mfa1 subunits (Umemoto and Hamada, 2003). Considering the heterogeneity of lipid A and fimbriae, *P. gingivalis* can induce different immune responses and even escape from immune surveillance and attack (Bostanci and Belibasakis, 2012).

## The Interaction Between Autophagy and Infection

Autophagy majorly plays a protective role in the elimination of intracellular bacteria. Upon infection, p62 or other autophagy receptors are recruited to invading ubiquitinated bacteria and delivered them to autophagosome (Zheng et al., 2009). Thurston found that pattern recognition or danger receptors, such as galectin 8 could label invading *Salmonella* as autophagy cargo and activate xenophagy by recruiting NDP52 (Thurston et al., 2012). Both autophagy receptor p62 and NDP52 could detect ubiquitin-coated bacteria in the cytosol and deliver them into autophagosomes for degradation (Thurston et al., 2009; Randow, 2011).

However, bacteria have evolved to manipulate the autophagic way to escape from immune attacks and utilize autophagy for nutrients (Randow and Munz, 2012; Bah and Vergne, 2017). For example, *L. pneumophila* could inhibit autophagy flux by cleaving syntaxin-17, a key SNARE protein implicated in autophagosome formation (Arasaki et al., 2017). Besides *L. pneumophila* could also inhibit the formation of autophagosomes by cleaving phosphatidylethanolamine-conjugated LC3 (Choy et al., 2012). The two mechanisms involved in the cleavage of autophagy machinery were respectively achieved by two specific proteases, Lpg1137 and RavZ (Choy et al., 2012; Arasaki et al., 2017). Canonical macroautophagy limits the growth of bacterial replication. But Steele et al. (Steele et al., 2013) found that *F. tularensis* could induce an Atg5-independent autophagy pathway which was beneficial for cytoplasmic *F. tularensis* growth by supplying nutrients in primary human monocytes. *F. tularensis* growth was significantly decreased by treatment with 3-MA and it was rescued by supplementing excess amino acids. Therefore, autophagy could provide amino acids that support *F. tularensis* growth (Steele et al., 2013).

In summary, the interaction between bacterial pathogens and the infected host cell determines the former’s survival or elimination. The involved mechanism varies in different bacterial and host cells, which are listed in **Table 1**.

**Table 1:** Summary of the mechanisms how the bacteria interact with autophagy.

Pathogen	Effector	Host	target	Mechanism of escaping from host autophagy attack	Reference
*Porphyromonas gingivalis*		HGE		Hide in ER-rich-double-membrane autophagosomal-vacuoles	(Lee et al., 2018)
				Sorted to Rab11 and RalA-positive recycling endosomes	(Takeuchi et al., 2011)
	Mfa1 fimbriae	DC		Activate mTOR and inhibit ULK1 to decrease autophagic activity	(Meghil et al., 2019)
			DC-SING	Hide in single membrane vesicles	(El-Awady et al., 2015)
		HACE		Hide in autophagosome	(Dorn et al., 2001)
*Mycobacterium tuberculosis*		Macrophage	ATPase	Inhibit phagosomes fuse with lysosome	(Sturgill-Koszycki et al., 1994)
	ESX-1	DC		Inhibit autophagosome fuse with lysosome	(Romagnoli et al., 2012)
Group A *Streptococcus* (M1T1)	SpeB	Human HEp-2 epithelial cells	NDP52, p62 and NBR1	Avoid ubiquitylation and recognition by the host autophagy marker LC3 and ubiquitin-LC3 adaptor proteins NDP52, p62 and NBR1	(Barnett et al., 2013)
*Legionella*	Birc1e/Naip5	Macrophage		Inhibit autophagosome fuse with lysosome	(Fortier et al., 2007)
	RavZ	BMDM	LC3-II	Inhibit the initiation of autophagy	(Choy et al., 2012)
*Shigella flexneri*	VirA	Hela	Rab1	Inhibit the early phagophore formation	(Dong et al., 2012)
	IcsB, VirG	BHK	ATG5	Inhibit the initiation of autophagy.	(Ogawa et al., 2005)
*Salmonella Typhimurium*	T3SS2		FAK/AKT	Inhibit the initiation of autophagy by AKT/mTOR signaling pathway.	(Casanova, 2017)
	SopF	Hela	VTPase-ATG16L1	Block the V-ATPase recruiting ATG16L1 onto bacteria-contain vacuole.	(Xu et al., 2019)
		epithelial cell	mTOR	Reactivation of mTOR by *Salmonella* Results in Autophagy Escape	(Tattoli et al., 2012)
*Listeria monocytogenes*	ActA	MDCK	Actin	Escape from autophagy by protein recruitment	(Yoshikawa et al., 2009)
	Listeriolysin O	mouse embryonic fibroblast		Phospholipase assists *Listeria monocytogenes* to escape autophagy.	(Py et al., 2007)
*Anaplasma phagocytophilum*	Ats-1	HEK 293	Beclin-1	Hijack the Beclin 1-Atg14L autophagy initiation pathway	(Niu et al., 2012)
*Helicobacter pylor*		gastric epithelial cell		Inhibit autophagosome fuse with lysosome	(Zhang L. et al., 2018)

## The Interaction Between Autophagy and *P. gingivalis* Infection Affects the Inflammation

As a major pathogen of periodontitis, *P. gingivalis* can manipulate the host’s innate immune response through different mechanisms, ultimately leading to the loss of periodontal supporting tissues, including the periodontal ligament and alveolar bone (Kinane et al., 2017). *P. gingivalis* and its virulence factors are recognized by pattern recognition receptors (PRRs), such as toll-like receptors (TLRs) and nod-like receptors (NLRs) (Fan et al., 2015; Watanabe et al., 2020). This process can not only trigger an inflammatory response but also induce autophagy to achieve the purpose of clearing *P. gingivalis*. However, *P. gingivalis* has developed diverse mechanisms to avoid recognition, inhibit the autophagic pathway, or may hijack the autophagosome (Wu and Li, 2019). The aberrant of autophagy leads to the imbalance between the pro-inflammatory and anti-inflammatory processes. Consequently, whether autophagy plays a protective role in the *P. gingivalis*-induced inflammation is determined by the interaction between autophagy and *P. gingivalis* infection.

### Autophagy Defends Against *P. gingivalis* to Limit Inflammation

*P. gingivalis* invades the host cells by cytoskeletal rearrangement to form phagocytose, after which autophagy starts up and promotes bacteria elimination by fusing with lysosomes (Hu et al., 2020). In addition, autophagy acts as an innate immune response and plays a protective role in combating *P. gingivalis* infection and downregulating inflammatory response (Park et al., 2017; Liu et al., 2018).

#### Autophagy Plays a Protective Role in Periodontal Tissue Cells

Autophagy can protect host cells from apoptosis when periodontal tissues are subjected to *P. gingivalis* and its virulence factors. The possible involved mechanism is that the activation of autophagy may suppress apoptosis. For example, compared with healthy people, the expression levels of ATGs such as LC3, Beclin-1, Atg7, and Atg12 are higher in periodontal ligament tissue cells (PDLSCs) of periodontitis patients. However, the apoptotic protein caspase-8 expresses less (An et al., 2016). Coincidentally, another research found that the expression of Atg12 and the conversion of LC3-I to L3-II were increased in human gingival fibroblasts (HGFs) treated with *P. gingivalis* LPS (Bullon et al., 2012). Pro-inflammatory cytokines, such as TNF-α and IL-1β secretion could be limited by *P. gingivalis*-induced autophagy (Liu et al., 2018). Inhibiting autophagy with 3-Methyladenine (3-MA) increased the apoptosis rate in infected cells (Bullon et al., 2012), which indicated that autophagy might protect host cells from apoptosis in inflammation (Bullon et al., 2012; Liu et al., 2018).

Besides, autophagy can also reduce the secretion or increases the degradation of inflammatory factors. Chang *et al.* (Chang et al., 2013) found that butyrate, a kind of metabolic production of *P. gingivalis*, could impair cell cycle progression and induce inflammation *via* the generation of ROS in HGFs. However, *P. gingivalis* LPS treatment could also induce an autophagic response in human cultured keratinocyte cells (HaCaT) *via* increased ROS (Hagio-Izaki et al., 2018). The expression of LPS binding protein and TLRs increased, resulting in the co-localization of *P. gingivalis* with autophagosomes (Hagio-Izaki et al., 2018). Antibacterial peptide LL-37 could reduce the number of live *P. gingivalis* in HaCaT in a dose-dependent manner *via* xenophagy. Treatment with 3-MA weakened the inhibitory effect of LL-37 on the intracellular *P. gingivalis* (Yang et al., 2020). The findings indicated that autophagy was beneficial to the defense against the *P. gingivalis* internalized in HaCaT. Furthermore, inhibiting autophagy could enhance inflammation. Huang et al. (Huang et al., 2020) found high glucose environment could disrupt the autophagy lysosomal pathway (ALP) *via* a special subunit on the ATPase transmembrane (ATP6V0C), consequently enhancing the expression of IL-1β in HGECs.

Moreover, autophagy also plays a protective role by modulating the proangiogenesis ability to protect cells under the inflammatory environment. Wei et al. (Wei et al., 2018) found that proangiogenesis cytokine, angiogenin (Ang) expression is higher in inflammatory human periodontal ligament stem tissues (PDLSCs) and more tube formation was observed in endothelial cells pretreated with TNF-α and IL-1β. Rapamycin or Beclin-1 overexpression-induced autophagy can upregulate the expression level of Ang in PDLSCs, while knockdown of Beclin-1 downregulates its expression.

Consequently, when periodontal tissue cells are stimulated by *P. gingivalis* or its virulence factors, autophagy plays a protective role in fighting against the pathogens and decreasing the inflammatory injury. The involved mechanism may be that autophagy can decrease the intracellular *P. gingivalis*, reduce the secretion of inflammatory factors, inhibit host cell apoptosis death, and promote angiogenesis ability.

#### Autophagy Plays a Dual Role in Osteoclasts Differentiation and Bone Resorption

As an important indicator for the diagnosis of periodontitis, alveolar bone resorption is largely irreversible (Armitage, 2004). *P. gingivalis* infection could accelerate the alveolar bone pathological resorption by ROS generation and autophagic activity (Waddington et al., 2000; Tokutomi et al., 2015; Huck et al., 2020). Autophagy plays a dual role in osteoclast differentiation and bone resorption during periodontitis. DeSelm et al. (DeSelm et al., 2011) elucidated that osteoclasts degrade bone by directionally secreting lysosomal enzymes to a complex structure named ruffled border. The ruffled border is the product of the fusion of secretory vesicles to the bone-apposed plasma membrane. Lysosomal fusion with the plasmalemma results in the release of matrix-degrading molecules such as cathepsin K into the extracellular space to digest the bone matrix (DeSelm et al., 2011). However, PE-conjugated LC3 system and ubiquitin systems are essential for generating the osteoclast ruffled border, the secretory function of osteoclasts, and bone resorption (DeSelm et al., 2011). The research found that Syndecan 4 (SDC4), a member of the syndecan family, was highly expressed in the experimental periodontitis rat model (Li J. et al., 2021). While SDC4 silencing inhibits osteoclast differentiation and SDC4 overexpression enhanced osteoclast differentiation and autophagy induced by receptor activator of NF-κB ligand (RANKL). However, inhibiting autophagy with 3-MA abolished osteoclast differentiation which was enhanced by SDC4, indicating that autophagy might promote the osteoclast differentiation during periodontitis (Li J. et al., 2021).

On the other hand, autophagy also plays a negative role in osteoclast differentiation. Song et al. (Song et al., 2019) found that osteoclast differentiation and mRNA expression of osteoclast−specific genes were enhanced in bone marrow macrophages stimulated by IL-17A. Inhibition of autophagy with 3−MA attenuated the IL−17A−induced osteoclast differentiation. Tang et al. (Tong et al., 2018) found that autophagy plays an essential role in the process *via* AMPK/mTOR/p70S6K signaling pathway. Inhibiting autophagy with chloroquine (CQ) attenuated the OPG’s inhibition of osteoclast differentiation and bone resorption. Besides, autophagy also plays an important role in orthodontic tooth movement (Li Y. et al., 2021). Autophagy activity enhanced on the compression side of the tooth, which was associated with osteoclast recruitment and inflammatory cytokine expression (Li Y. et al., 2021). Treatment with rapamycin, an autophagy promotor, resulted in less bone resorption and tooth movement, suggesting that autophagy downregulated the inflammatory response and osteoclast differentiation during orthodontic tooth movement (Li Y. et al., 2021). To sum up, regulating autophagy activity may be an effective way to protect alveolar bone from excessive resorption in periodontitis.

#### Autophagy Plays a Protective Role in Antigen-Presenting Cells

*P. gingivalis* can invade antigen-presenting cells (APCs) such as macrophage and dendritic cells (DCs) and elevate autophagy activity. Autophagy can also inhibit inflammation by the elimination of *P. gingivalis* and degradation of inflammatory factors in APCs.

As for macrophages, Park et al.. (Park et al., 2017) preliminarily elucidated the link among bacteria, autophagy, and inflammation in the model of *P. gingivalis* infection in THP-1 cells. The infection promoted the secretion of mature IL-1β through activation of NLRP3 inflammasomes and downstream caspase-1. Similarly, *Aggregatibacter actinomycetemcomitans* (*A. actinomycetemcomitans*), another important pathogen of periodontitis, can also promote IL-1β maturation through the activation of AIM2 (absent in melanoma 2) inflammasome (Lee et al., 2020). The induction of AIM2 or NLRP3 inflammasomes could trigger autophagosome formation, which is dependent on the presence of the inflammasome sensor (Shi et al., 2012). The inflammasomes could be ubiquitinated and then recruit the ubiquitin-binding protein adaptor p62. With its assistance, the inflammasomes were delivered into autolysosome for degradation (Shi et al., 2012). Besides, both *P. gingivalis* and *A. actinomycetemcomitans*-induced autophagy in THP-1 cells inhibited the production of intracellular IL-1β and ROS by promoting bacterial internalization *via* phagocytosis into the macrophages. This therefore restricted excessive inflammatory response (Park et al., 2017; Lee et al., 2020). Besides, recent studies found two compounds, lipoxin A4 and vitamin D could promote the autophagic activity upon *P. gingivalis* infection in macrophages (Niu et al., 2020; Zhao et al., 2021). Lipoxin A4 inhibited NLRP3 inflammasome and downstream caspase-1 and IL-1β in *P. gingivalis* LPS-infected RAW264.7 by promoting autophagy. This action was related to the phosphorylation of NF-κB (Zhao et al., 2021). Calcitriol could significantly enhance the colocalization of *P. gingivalis* with autophagosome and lysosome markers in U937-derived macrophages, which indicated that the autophagy process could degrade live *P. gingivalis* (Niu et al., 2020). In summary, the above studies elucidated the role of autophagy in suppressing *P. gingivalis*-induced inflammation in macrophages. On the one hand, autophagy inhibits the production and promotes the degradation of AIM2 and NLRP3 inflammasomes. On the other hand, autophagy promotes bacterial internalization through phagocytosis and limits excessive inflammatory responses.

The autophagic activity in DCs was different from macrophages upon infection. *P. gingivalis* recognizes different PRRs on the surface or in the cytoplasm of DCs, which can trigger different responses (Xie et al., 1997; den Dunnen et al., 2009). The PRRs included TLRs and C-type lectin receptors such as DC-SIGN. The activation of TLR2 by FimA fimbriae could inhibit Akt phosphorylation, whereas the activation of DC-SIGN by Mfa-1 fimbriae could promote Akt phosphorylation (El-Awady et al., 2015; Meghil et al., 2019). Phosphorylated Akt could phosphorylate and activate mTORC1, ultimately inhibiting its downstream effector ULK1 to decrease autophagic activity. The interaction between DC-SIGN and Mfa-1 *P. gingivalis* strains induced lower intracellular killing and higher intracellular content of *P. gingivalis*. Moreover, Mfa-1 *P. gingivalis* was majorly wrapped in single-membrane vesicles, where it survived intracellularly (El-Awady et al., 2015). The finding indicated that Mfa-1 *P. gingivalis* engaged with DC-SIGN promoted the evasion of xenophagy. However, the activation of TLR2 cannot escape the autophagic attack. In addition, transcription factor forkhead box O1 (FOXO1) was phosphorylated and inactivated by Akt in DCs infected with Mfa-1 positive strains *Pg*381, causing FOXO1 to relocate to the cytoplasm (Meghil et al., 2019). FOXO1 deletion could inhibit the migration of DCs to lymph nodes and reduce the capacity of DCs to promote the formation of plasma cells and decrease the production of bacteria-specific antibodies (Xiao et al., 2015). Thus, the inactivation of FOXO1 promotes the survival of intracellular *P. gingivalis.*

#### Autophagy Plays a Protective Role in Other Systematic Inflammatory Diseases

The invasion of oral *P. gingivalis* into blood circulation is related to several systemic inflammatory diseases. For example, *P. gingivalis*-induced periodontitis could increase the risk of atherosclerosis, and *P. gingivalis* could also be detected in human atherosclerotic plaques (Kozarov et al., 2005). Research found that *P. gingivalis* could induce human umbilical vein endothelial cells (HUVECs) ER stress-mediated apoptosis and autophagy. The autophagic response protects HUVEC from apoptosis and silencing of LC3 with siRNA significantly increased *P. gingivalis*-induced apoptosis (Hirasawa and Kurita-Ochiai, 2018).

Infection with *P. gingivalis* can also accelerate the development and progression of non-alcoholic fatty liver disease (NAFLD) (Yoneda et al., 2012), a disease characterized by intracellular accumulation of lipid droplets in hepatocytes. Zaitsu et al. (Zaitsu et al., 2016). found that internalized *P. gingivalis* was localized in autophagosomes or lysosomes rather than lipid droplets in HepG2 hepatocytes, indicating that *P. gingivalis* could not use the lipid droplets for nutritional purposes. However, lipid droplets could affect the formation of autolysosomes for degradation of *P. gingivalis* and increase the existence of *P. gingivalis* in the cells at an early phase of infection. Thus, delayed elimination of *P. gingivalis* prolonged the inflammation and cellular damage, which raise the risk for the development of NAFLD (Zaitsu et al., 2016).

### *P. gingivalis* Inhibit or Escape Autophagy for Survival and may Promote Inflammation

The above researches preliminarily revealed that autophagy functioned as a defender to protect host cells against *P. gingivalis* in various periodontal tissue cells, osteoclasts, antigen-presenting cells, and other tissue cells. However, *P. gingivalis* can also interfere with the process of autophagy to escape from the host immune attack and then the inflammation shall persist.

#### *P. gingivalis* can Hide in Autophagosome for Survival

*P. gingivalis* can escape from autophagy to avoid degradation by hindering the combination of autophagosomes with lysosomes. In this situation, *P. gingivalis* could colonize and proliferate in a vacuole encapsulated by a monolayer membrane after its invasion. The autophagosome with a monolayer membrane is modified by *P. gingivalis* and cannot fuse with lysosomes to form autolysosomes, exerting its degradation effect. Instead of the lysosomal marker protein LAMP-1, only rough ER protein attaches to the surface around the vacuole (Dorn et al., 2001). Lee et al. (Lee et al., 2018). found *P. gingivalis* adhered to the surface of GECs and was trafficked into the ER-rich autophagosomes through lipid rafts. After initially localized in early endosomes in GECs, some *P. gingival* entered the late endosomes where they were then sorted to lysosomes for degradation, some were delivered to Rab11 and RalA positive recycling endosomes for exocytosis while others escaped from endosomes to autophagosomes (Takeuchi et al., 2011; Amano et al., 2014). Hiding in autophagosomes, *P gingivalis* could utilize the substances such as protein for its survival. Inhibition of autophagy by 3-MA or Atg5 siRNA significantly decreased the survival of internalized *P. gingivalis* in HGECs (Belanger et al., 2006; Lee et al., 2018). *P. gingivalis* could colonize and replicate in GECs by inhibiting pro-apoptotic Bad through Akt to facilitate its long-term survival (Yao et al., 2010).

#### *P. gingivalis* Inhibit Autophagy Through LAP

Epidemiological studies have also linked PD to age-related macular degeneration (AMD) (Winning and Linden, 2017). Recent researches reported that *P. gingivalis* invasion and autophagy evasion might contribute to the pathogenesis of retinal degenerative diseases (Arjunan et al., 2020). This study showed that *P. gingivalis* could invade the human-retinal pigment epithelial (ARPE) cells and was able to escape from the autophagic vesicles and traffic into single membrane structures, freely occupying the cytoplasm of ARPE cells (Arjunan et al., 2020). The underlying mechanism may be associated with MREG-dependent LC3-associated phagocytosis (LAP). Lysosome maturation required the binding of LC3 to melanoregulin (MREG), and the MREG-mediated LC3-dependent degradation pathway was an essential process for *P. gingivalis* clearance (Martinez et al., 2011; Frost et al., 2015). Frost et al. (Frost et al., 2015) observed the co‐localization of LC3 and MREG in GECs of patients with severe periodontitis, but not in mild periodontitis or healthy people. The result showed that the expression of LC3, Beclin-1, and MREG in periodontitis was less than that in healthy people. It is speculated that *P. gingivalis* may affect the expression of MREG and LC3, and inhibit the normal autophagic process in HGECs of periodontitis patients.

#### The Mechanism of Inhibiting Autophagy by *P. gingivalis* Varies in Different Strains

As mentioned above, *P. gingivalis* expressed two forms of fimbriae and could interact with different PRRs on the host cell, affecting the fate of engulfed bacteria. Both long and short fimbriae were critical for the initiation of inflammatory responses and alveolar bone loss in periodontitis (Umemoto and Hamada, 2003). However, the interaction between Mfa-1 positive strains Pg381 with DC-SIGN could promote Akt phosphorylation and decrease the expression of LC3-II, Rab5, and LAMP1, thus inducing lower intracellular killing of *P. gingivalis* in DCs (El-Awady et al., 2015). However, the activation of TLR2 by FimA positive strains cannot escape from autophagic attacks. A similar phenomenon was found in *Pg*381 strain-infected human coronary artery endothelial (HCAE) cells. The survival rate of intracellular *P. gingivalis* decreased when HCAE cells were pretreated with 3-MA or wortmannin (Dorn et al., 2001). However, the pathogenic mechanisms varied in different *P. gingivalis* strains (Rodrigues et al., 2012; Blasi et al., 2016). (Rodrigues et al., 2012). compared different *P. gingivalis* strains (*Pg*W83, *Pg*A7436, *Pg*381, and *Pg*33277) and their abilities of adherence, internalization, and survival rate in HCAE cells. Both *Pg*W83 and *Pg*381 were trafficked through the autophagic pathway, but only *Pg*381 could survive, depending on the autophagic pathway. Furthermore, *Pg*381 strain infected-HACE cells resulted in the highest production of pro-inflammatory cytokines IL-6, IL-8, and TNF-a.

## The Possible Mechanisms Involved in the Interaction Between Autophagy and *P. gingivalis*-Induced Inflammation

**Figure 2:**
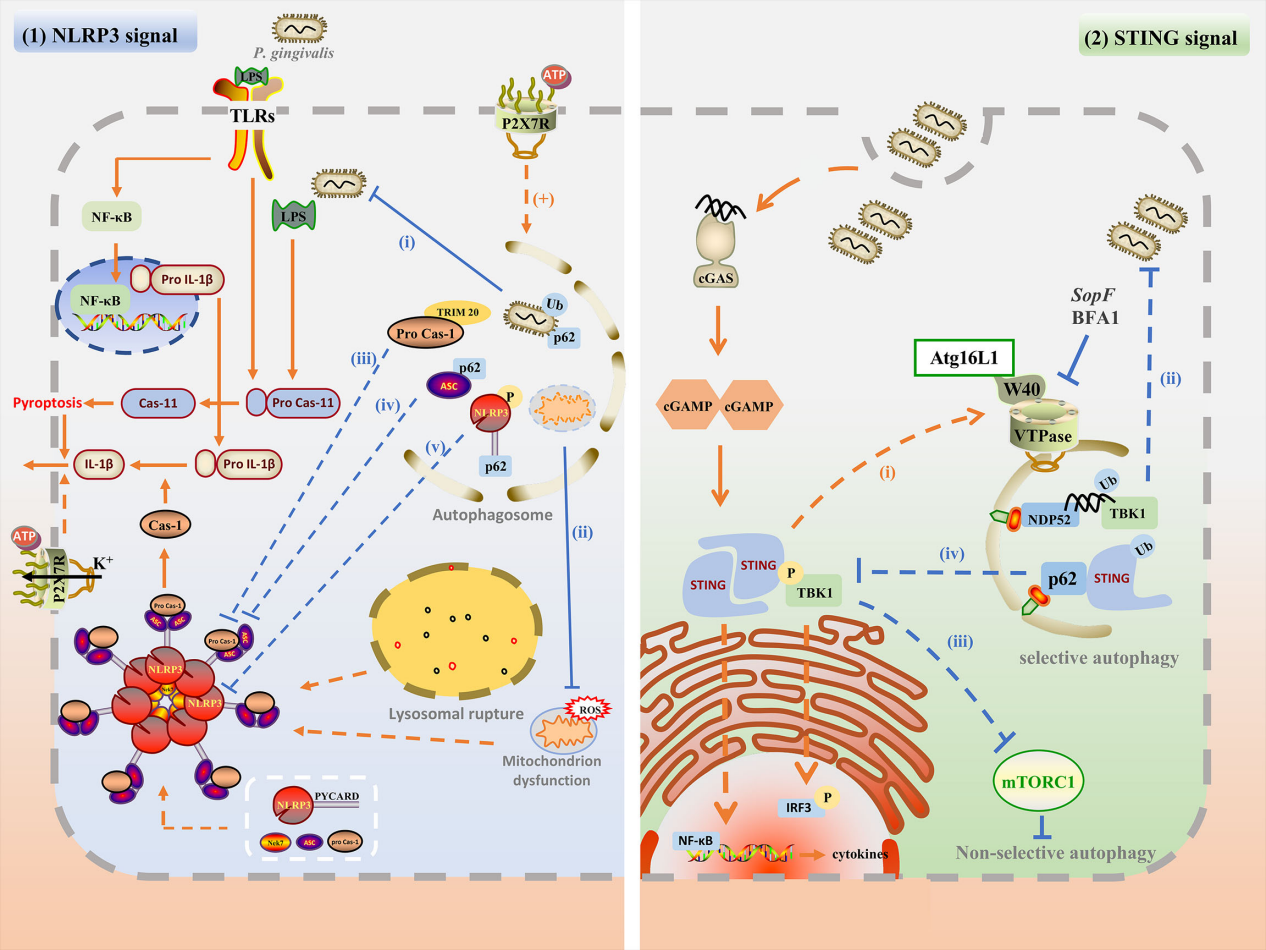
The possible mechanisms involved in the interaction between autophagy and *P. gingivalis*-induced inflammation. (1) Upon *P. gingivalis* infection, NLRP3 inflammasome can be activated by TLR/NF-κB signal, ROS generation, P2X7R and potassium efflux, and lysosomal rupture. The assembly of NLRP3 inflammasome promotes caspase-1 maturation as well as active IL-1β secretion, and pyroptosis. Autophagy may inhibit NLPR3 inflammasome by degrading intracellular *P. gingivalis*(i), dysfunctional mitochondrion (ii), phosphorylated NLRP3-PYCARD (iii), ubiquitinated ASC (iv), and pro-caspase-1 (v). Besides, P2RX7 activation may promote the autophagy flux, which is beneficial for bacterial degradation. (2) Autophagy may regulate the cGAS-cGAMP-STING pathway. cGAS may detect *P. gingivalis* infection by its dsDNA, and activate downstream cGAMP and STING. STING recruits and phosphorylates TBK and activated TBK1 kinase phosphorylates downstream IRF3. STING also activates NF-κB to induce IFN and other cytokines. Activated STING may recruit Atg16L1 and interact with VTPase *via* its WD40 domain to promote autophagy for degradation of *P. gingivalis* (i). Cytosolic dsDNA may be directly targeted for autophagosome *via* ubiquitin, TBK1, and NDP52-mediated xenophagy for degradation (ii). Besides, STING and TBK1 kinase may promote non-selective autophagy by inhibiting mTORC1 (iii). TBK1 kinase may also recruit and phosphorylates p62, enhancing the affinity of p62 with ubiquitinated STING and promoting selective autophagy.

The above research summarized the different interactions between autophagy and *P. gingivalis*, which may affect inflammation. On the one hand, autophagy inhibits inflammation by targeting the intracellular pathogens for degradation; on the other hand, *P. gingivalis* can take advantage of autophagosomes to escape from the host attack and prolong the inflammation. Autophagy can also interact with inflammation directly. Therefore, the mechanisms of the specific interaction among autophagy, *P. gingivalis*, and inflammation are significant in exploring the pathogenesis and cure of periodontitis. Based on *P. gingivalis* and other similar pathogens infection, this chapter summarized the complex interaction through the NLRP3 inflammasome pathway and cGAMP-cGAS-STING signaling pathway. The possible mechanisms involved in the interaction between autophagy and *P. gingivalis*-induced inflammation are plotted in **Figure 2.**

### Autophagy may Negatively Regulate Inflammation By Interacting With the NLRP3 Inflammasome Pathway

#### NLRP3 Inflammasome Pathway Introduction

NLRP3 inflammasome is a multiprotein complex that contains adapter protein apoptosis-associated speck-like protein (ASC) and procaspase-1. Its biochemical function is to activate caspase-1, which leads to the maturation of IL-1β and IL-18 (Shao et al., 2015; He et al., 2016a). Recent studies found a serine/threonine kinase named NEK7, which is bound to the leucine-rich repeat domain of NLRP3. NEK7 is also an essential mediator of NLRP3 activation (He et al., 2016b; Shi et al., 2016).

The NLRP3 inflammasome pathway activation involves two steps. In the initiating step, TLRs sense the invading *P. gingivalis* outside the cell and in intracellular endosomes and lysosomes (Medzhitov, 2001; Zhou et al., 2005; Zhang J. et al., 2018), leading to activation of NF-κB-mediated signaling, which in turn induces the transcription of NLRP3, pro-IL-1β (Bauernfeind et al., 2009; Hornung and Latz, 2010). The second step of inflammasome activation is the assembly of NLRP3, ASC, and pro-caspase-1 into a complex (Petrilli et al., 2007; Shao et al., 2015; Jo et al., 2016). *P. gingivalis* LPS also engages in the non-canonical inflammasome pathway (Ding et al., 2020; De Andrade et al., 2021), in which caspase-4 (known as caspase-11) is activated, promoting pyroptosis through cleavage of the pore-forming protein gasdermin D, resulting in maturation and release of IL-1β (Kayagaki et al., 2011; Hagar et al., 2013; Kayagaki et al., 2013). Different from the canonical NLRP3 inflammasome pathway, TLR4 senses the extracellular *P. gingivalis* LPS while caspase-4/11 senses the cytosolic LPS (Kayagaki et al., 2013; Downs et al., 2020; Xie et al., 2020)

The molecular mechanisms involved in NLRP3 inflammasome activation include mitochondrial ROS generation (Sorbara and Girardin, 2011; Heid et al., 2013), lysosomal rupture (Heid et al., 2013), pore formation, and potassium (K+) efflux (Petrilli et al., 2007). The P2X7 receptor is an ion channel activated by extracellular adenosine triphosphate (eATP) that induces IL-1β secretion through NLRP3 inflammasome activation (Park et al., 2014; Di Virgilio et al., 2017; Almeida-da-Silva et al., 2019).

#### Autophagy Plays a Protective Role in Inflammation by Interacting With the NLRP3 Inflammasome Pathway

Recent research reported that autophagy could suppress the activation of NLRP3 inflammasome. Enhancing autophagy with rapamycin reduced the expression of ASC as well as caspase-1 p20, and decreased the secretion of IL-1β, which in turn attenuated myocardial ischemia-reperfusion injury in diabetic rats (Zhang et al., 2020). Besides, Atg16L1-deficient macrophages produce high amounts of cleaved caspase-1 and IL-1β following stimulation of LPS (Saitoh et al., 2008). However, the mechanism of how autophagy suppressed the activation and assembly of inflammasomes upon *P. gingivalis* infection is intricate. It is supposed that this may be linked to clearing pathogens, degrading inflammasome components, and interrupting the molecular mechanism (Cao et al., 2019).

Xenophagy effectively limits intracellular bacterial survival and replication. Cytoplasmic bacteria, such as *Salmonella. Typhimurium* (*S. Typhimurium*), can be enveloped by ubiquitin proteins and recognized by autophagy receptors, such as p62, NDP52, OPTN, and TAX1BP1. With these receptors’ help, cytoplasmic bacteria are targeted to autophagosome (Thurston et al., 2009; Wild and Farhan, 2011; Tumbarello et al., 2015; Lee et al., 2022). In addition to ubiquitin, galectin-8, a cytosolic lectin, detects *S. Typhimurium* invasion by binding host glycans after being exposed to damaged *Salmonella*-containing vacuole (SCV). Galectin-8 can recruit NDP52 transiently and then NDP52 are recruited in a ubiquitin-dependent manner to activate xenophagy (Thurston et al., 2012). Although xenophagy machinery of targeting bacteria for degradation is well established, the ubiquitination mechanism and how autophagy receptor senses bacterial, especially *P. gingivalis*, are not fully clear. Only p62 and NDP52 receptors are demonstrated to bind to cytosolic *P. gingivalis* so far (Lee et al., 2018), and whether OPTN and TAXIBP1 or other ubiquitin adaptor proteins could target *P. gingivalis* to autophagosome is not clear. However, infecting *P. gingivalis* can be wrapped in ER-rich-double-membrane autophagosomal-vacuoles for survival. Inhibiting autophagy with 3-MA or ATG5 siRNA significantly reduced the viability of intracellular *P. gingivalis*. Besides, p62 and NDP52 receptors only bind to cytosolic free *P. gingivalis* while the vacuolar-*P. gingivalis*, which reveals a novel mechanism of *P. gingivalis* survival from xenophagy (Lee et al., 2018).

The activation of NLRP3 inflammasome and its components could be inhibited by autophagy. During the priming step of NLRP3 inflammasome activation, autophagy can inhibit NF-κB activation and the expression of pro-IL-1β (Wu et al., 2020), but the mechanism is not clear. During the process of inflammasomes’ assembly, the ubiquitination or phosphorylation modification of components can trigger selective autophagy. Spalinger et al. (Spalinger et al., 2017) found that only phosphorylated NLRP3 is found in autophagosomes and NLRP3 lacking phosphorylation sites cannot be recruited to phagophores upon inflammasome activation. Phosphorylated NLRP3 binds to p62 and is recruited into phagophores for degradation, which is dependent on NLRP3-PYCARD interaction (Spalinger et al., 2017). NLRP3 does not interact with SQSTM1in PYCARD-deficient cells, indicating that PYCARD may be the tag recognized by p62 for selective autophagy. The specific interaction and mechanism are worth further exploration. In addition to NLRP3, ASC under ubiquitination can recruit p62. With its assistance, ASC is delivered to autophagosomes for degradation in THP-1 treated with LPS and ATP (Shi et al., 2012). Besides to ubiquitination or phosphorylation modification-mediated selective autophagy, TRIMs family protein-mediated a highly exact process termed precision autophagy also plays an important role in the inactivation of NLRP3 inflammasome (Kimura et al., 2016). TRIM family proteins functioned as receptor-regulator proteins, both recognized targets and mediated autophagy. Different from selective autophagy, TRIMs could recognize targets through a direct protein-protein binding way without ubiquitin or galectin intermediates. And TRIMs function as platforms for the assembly of the core regulators of autophagy, such as the ULK1 complex or Atg16L1 complex (Kimura et al., 2016). For example, TRIM20 directly recognizes NLRP3 inflammasome and its component procaspase-1 and presents them for autophagic degradation (Kimura et al., 2015). The receptor and regulatory features enable TRIMs to mediate the inflammasome degradation *via* a highly exact process termed precision autophagy.

Autophagy also interacts with the molecular mechanisms involved in NLRP3 inflammasome activation, such as ROS clearance and P2X7 receptor. It has been demonstrated that *P. gingivalis* LPS-induced cytosolic ROS plays an important role in the pathogenesis of periodontitis (Chapple and Matthews, 2007; Golz et al., 2014). Mitophagy could preserve mitochondrial integrity and eliminate ROS through the degrading of damaged mitochondria, thereby reducing NLRP3 inflammasome activation (Lin et al., 2019). P2X7 receptor expression is enhanced in the *P. gingivalis* infection model, which is essential for NLRP3 activation promotes. The activation of the P2X7 receptor could promote *P. gingivalis* elimination in macrophages (Almeida-da-Silva et al., 2019). The possible mechanism may be the activation of P2RX7 can activate the AMPK/ULK1 pathway to promote autophagy flux for degradation of intracellular pathogens (Dong et al., 2021).

### Autophagy may Regulate Inflammation by Interacting With the cGAS-cGAMP-STING Pathway

It is demonstrated that the cGAS-cGAMP-STING pathway-mediated interferon (IFN) activation plays an essential role in the resistance to DNA viral infection (Cai et al., 2014). However, emerging evidence has linked the cGAS-cGAMP-STING pathway with autophagy and both pro-inflammation and anti-bacteria effects upon infection have been reported (Gui et al., 2019).

As for pro-inflammatory activity, cyclic GMP-AMP synthase (cGAS) detects infections by binding to cytosolic DNA from bacteria or viruses (Sun et al., 2013; Collins et al., 2015). Upon recognition of double-stranded DNA, cGAS produces cyclic GMP-AMP (cGAMP), a second messenger for signal transduction (Kato et al., 2017). cGAMP binds to the adaptor protein STING, which promotes STING dimerization and translocation from the ER to perinuclear structures or Golgi apparatus (Ishikawa and Barber, 2008). STING recruits and activates TANK binding kinase 1 (TBK1), phosphorylating the IFN regulatory factor 3 (IRF3) (Abe and Barber, 2014; Liu et al., 2015). STING also activates NF-κB, which functions together with IRF3 to lead to the production of type 1 IFN and other inflammatory cytokines (Shu and Wang, 2014).

As for anti-bacteria activity, the activation of the cGAS-STING pathway and TBK1 kinase can lead to ubiquitin-mediated xenophagy for the degradation of cytosolic dsDNA and bacteria (Watson et al., 2012; Watson et al., 2015; Gui et al., 2019). In addition to activating transcriptional responses, dsDNA is also a potent inducer of autophagy. Waston et al. (Watson et al., 2012) transfected plasmid dsDNA into LC3-GFP BMDMs and found that ubiquitin, TBK1, and NDP52 all colocalized to DNA puncta after transfection, indicating that cytosolic dsDNA was directly targeted for autophagosome *via* ubiquitin-mediated xenophagy, in which STING played an essential role. On the other hand, Collins et al. (Collins et al., 2015) found that *M. tuberculosis* infection-induced cGAS -STING pathway activation in THP-1. The absence of cGAS or STING significantly reduced the colocalization of LC3-II with *M. tuberculosis*, which was more permissive for *M. tuberculosis* survival. Besides to selective autophagy, TBK1 also promotes non-selective autophagy by suppressing the mTORC1 activity (Hasan et al., 2017). However, bacteria have involved the mechanism of escaping STING-induced autophagy. During the infection of *Salmonella*, STING-induced autophagy is mediated by recruiting Atg16L1 to interact with the vacuolar ATPase (VTPase) *via* its WD40 domain. The process can be blocked by bafilomycin A1 (BFA1), an autophagy inhibitor, and *SopF*, a T3SS effector from *Salmonella*, for they can bind to and inhibit the V-ATPase (Xu et al., 2019; Fischer et al., 2020). Furthermore, WIPI2-deficient cells showed less LC3 lipidation after treatment with cGAMP, indicating that Atg16L1 is essential in STING-induced autophagy. However, ULK1 and Beclin-1 showed less influence on STING-induced autophagy (Gui et al., 2019). Although the cGAS-STING pathway plays an important protective role in the elimination of bacteria by autophagy, the specific mechanism of how the cGAS-STING pathway interacts with the autophagy pathway remains unclear.

Interestingly, the association between the cGAS-STING pathway and autophagy is like a negative feedback loop. Once the cGAS-STING pathway is activated to cGAS, TBK1 kinase recruits and phosphorylates p62, enhancing the affinity of p62 with ubiquitinated STING. As a consequence, STING is degraded through selective autophagy, attenuating the cGAS-cGAMP-STING pathway response (Prabakaran et al., 2018).

However, similar studies of the cGAS-cGAMP-STING pathway are hardly found in the *P. gingivalis* infection model. Combining the mechanism of how *P. gingivalis*-induced autophagy and escape from autophagy with the cGAS-cGAMP-STING pathway, we make a summary as follows. On the one hand, internalized *P. gingivalis* and its LPS may activate the NLRP3 inflammasome, promoting Caspase-1 and IL-1β maturation and secretion. *P. gingivalis* dsDNA may activate the cGAS-cGAMP-STING pathway (Wan et al., 2020).On the other hand, the non-selective autophagy and LAP are like a defender fighting against the internalized *P. gingivalis*. This process is similar to innate immunity, resulting in the majority of the bacteria being delivered into the autolysosome for degradation. However, some *P. gingivalis* can escape from autophagy degradation by inhibiting the initiation and development of non-selective autophagy, reducing the fusion of the autophagosome with lysosome and hiding into the autophagosomal-vacuoles. In this condition, STING-induced autophagy and ubiquitination-mediated xenophagy may target the remaining *P. gingivalis* for degradation. This process resembles adaptive immunity. Popularly speaking, STING pathway-mediated autophagy is like another branch of the autophagy signaling pathway, but they can all end with the formation of autolysosomes for the degradation of pathogens.

## Summary

There is no doubt that the interaction between autophagy and *P. gingivalis* accounts for the balance of pro-inflammation and anti-inflammation to some extent, providing evidence for the possible mechanism of the pathogenesis of numerous inflammatory diseases, including periodontitis. In this review, we elaborate that autophagy majorly plays a protective role in defense against *P. gingivalis* infection and mediates *P. gingivalis* clearance to inhibit its inflammatory process. Besides, autophagy also interacts directly with the inflammatory processes, such as the NLPR3 inflammasome and cGAS-STING pathway, to restrict excessive inflammation (**Figure 2**). However, *P. gingivalis* and other bacteria have evolved the mechanism to escape or even hijack autophagy (**Table 1**), which may be unfavorable to the maintenance of host homeostasis. Therefore, it is of utmost importance to understand the molecular mechanisms of the interaction among autophagy, *P. gingivalis*, and inflammation. Such mechanisms ensure the normal anti-bacterial response that avoids excessive inflammatory damage and favors the maintenance of tissue homeostasis.

However, given the recent advanced researches on the interaction between autophagy, *P. gingivalis*, and inflammation, there is still a lack of critical evidence to elucidate the specific mechanism. 1) How *P. gingivalis* or other bacteria are modified and where are the accurate modification sites. 2) The specific mechanism of how *P. gingivalis* or other bacteria escape from autophagy is unspecific in most studies. The mechanism may be associated with the bacterial mutant virulence factors, host cell and receptors, and the specific interaction between the factors with the autophagy pathway. Precise interaction targets in the mechanism of bacteria capturing and escaping process are essential but still unclear. 3) The research on the anti-bacterial role of the cGAS-STING pathway and its interaction with autophagy and inflammation is still unclear and worth further exploration. 4) The evidence that autophagy plays a protective role in inflammatory disease is insufficient. Whether autophagy interacts directly with inflammation, such as the NLRP3 inflammasome pathway or other inflammatory factors is still unclear in *P. gingivalis* infection. On the other hand, there are few researches focused on the role of inflammation in autophagy. Excessive inflammation may interrupt the autophagy activity and in turn, forms a negative feedback effect on inflammation.

## Author Contributions

Writing-original draft preparation, SK; writing - review and editing, SK, P-HD, AD; supervision, P-HD, HW; project administration and funding acquisition, P-HD. All authors critically reviewed the manuscript before submission. All authors contributed to the article and approved the submitted version.

## Funding

This research was supported by the National Natural Science Foundation of China under grants No. 81870765 and 82170953.

## Conflict of Interest

The authors declare that the research was conducted in the absence of any commercial or financial relationships that could be construed as a potential conflict of interest.

## Publisher’s Note

All claims expressed in this article are solely those of the authors and do not necessarily represent those of their affiliated organizations, or those of the publisher, the editors and the reviewers. Any product that may be evaluated in this article, or claim that may be made by its manufacturer, is not guaranteed or endorsed by the publisher.
